# The role of the gut microbiota in regulating responses to vaccination: current knowledge and future directions

**DOI:** 10.1111/febs.17241

**Published:** 2024-08-05

**Authors:** Charné Rossouw, Feargal J. Ryan, David J. Lynn

**Affiliations:** ^1^ Precision Medicine South Australian Health and Medical Research Institute (SAHMRI) Adelaide Australia; ^2^ Flinders Health and Medical Research Institute Flinders University Bedford Park Australia

**Keywords:** antibody responses, COVID‐19, microbiome, microbiota, vaccination

## Abstract

Antigen‐specific B and T cell responses play a critical role in vaccine‐mediated protection against infectious diseases, but these responses are highly variable between individuals and vaccine immunogenicity is frequently sub‐optimal in infants, the elderly and in people living in low‐ and middle‐income countries. Although many factors such as nutrition, age, sex, genetics, environmental exposures, and infections may all contribute to variable vaccine immunogenicity, mounting evidence indicates that the gut microbiota is an important and targetable factor shaping optimal immune responses to vaccination. In this review, we discuss evidence from human, preclinical and experimental studies supporting a role for a healthy gut microbiota in mediating optimal vaccine immunogenicity, including the immunogenicity of COVID‐19 vaccines. Furthermore, we provide an overview of the potential mechanisms through which this could occur and discuss strategies that could be used to target the microbiota to boost vaccine immunogenicity where it is currently sub‐optimal.

## Graphical abstract

Vaccination plays a crucial role in mediating protection against infectious diseases, but immune responses to vaccination are highly variable and frequently sub‐optimal in vulnerable populations. Here, we review the latest preclinical and clinical evidence demonstrating that the gut microbiota plays a significant role in regulating immune responses to vaccination, discuss the potential mechanisms involved, and suggest future avenues of research in this field.

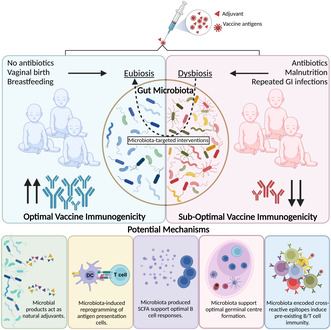

**Abbreviations**
AhR: aryl hydrocarbon receptor
BCG: Bacille Calmette–Guérin
COVID‐19: coronavirus disease 2019
DCs: dendritic cells
DTaP: diphtheria, tetanus, and acellular pertussis
FMT: faecal microbiota transplant
GALT: gut‐associated lymphoid tissue
GCs: germinal centres
HepB: hepatitis B
Hib: *Haemophilus influenzae* type b
HIV: human immunodeficiency virus
HMO: human milk oligosaccharides
HSA: human serum albumin
IgA: immunoglobulin A
IgG: immunoglobulin G
IL: interleukin
IPV: inactivated polio vaccine
LPS: lipopolysaccharide
MenB: meningococcal serogroup B
MenC: meningococcal serogroup C
MHC: major histocompatibility complex
mRNA: messenger RNA
MTIs: microbiota targeted interventions
NOD: nucleotide‐binding oligomerisation domain
ORV: oral rotavirus vaccine
OVA: ovalbumin
PCV: pneumococcal conjugate vaccine
PCV13: pneumococcal conjugate vaccine 13‐valent
pDCs: plasmacytoid dendritic cells
PRRs: pattern recognition receptors
RCTs: randomised controlled trials
SARS‐CoV‐2: severe acute respiratory syndrome coronavirus 2
SCFAs: short‐chain fatty acids
Tfh: T follicular helper
Th17: T helper 17
TLR: Toll‐like receptor

## Introduction

Edward Jenner's use of material from cowpox lesions to protect against smallpox in the late 18th century marked a pivotal moment in medical history, laying the groundwork for modern vaccination. Since then, global vaccination programs have saved millions of lives [1, 2] and led to the eradication or near‐eradication of diseases, including smallpox [3]. Furthermore, COVID‐19 vaccination programs prevented an estimated 20 million deaths in the first 12 months of the SARS‐CoV‐2 pandemic alone [2]. Vaccines primarily work by inducing the production of antigen‐specific antibodies that recognise and neutralise the pathogen targeted by the vaccine, and memory B cells that can rapidly respond when the targeted pathogen is encountered in future [4]. T cells are also crucial to the protection mediated by certain vaccines, such as the Bacille–Calmette–Guérin (BCG) vaccine against tuberculosis [5]. More recently, there has been renewed appreciation for the importance of T cell responses to vaccination as COVID‐19 mRNA vaccines have been shown to provide protection against severe disease caused by SARS‐CoV‐2 variants that can otherwise largely evade vaccine‐induced antibody responses [6, 7].

For reasons that are poorly understood, however, both B and T cell responses to vaccination show extreme inter‐individual variability and can be sub‐optimal or wane more quickly in certain vulnerable populations [8]. For example, the immunogenicity of many vaccines, that is the immune response provoked following administration, is blunted in infants, who thus require multiple booster doses to achieve levels of antibodies that are sufficient for protection [9]. Similarly blunted vaccine immunogenicity is also observed in the elderly. For example, the effectiveness of influenza vaccination can be as low as 30–50% in the elderly compared to 70–90% in younger adults [10]. This has led to the introduction of a high‐dose influenza vaccine to increase protection in older individuals [11]. Reduced vaccine immunogenicity has also been frequently observed in individuals from low‐ and middle‐income countries (LMICs) compared to those living in high‐income countries (HICs) [12]. Rotavirus vaccine efficacy at 12 months after oral immunisation, for instance, is estimated to be 94% in infants from HICs compared to less than 50% in infants from LMICs [13].

Many factors such as nutrition, age, sex, genetics, environmental exposures, and infections have all been reported to contribute to the variability in vaccine immunogenicity/effectiveness observed between different individuals and different populations [14]. Additionally, strong evidence accumulated over the past decade suggests that variation in the composition and function of the microbiota, particularly in the gut, is an important and targetable factor shaping optimal immune responses to vaccination. In early life, the gut microbiota starts out as a relatively low‐diversity community, predominantly consisting of *Enterobacteriaceae* and *Bifidobacteriaceae*. This community rapidly evolves, increasing in complexity in the first few months of life [15]. After weaning, the microbiota begins to more resemble the microbiota observed in adults and is characterised by a personalised and generally stable composition, except following perturbations such as antibiotic treatment [16, 17]. In adults, the gut microbiota is primarily composed of the phyla Bacilliota (formerly Firmicutes) and Bacteroidota (formerly Bacteroidetes) and is largely shaped by dietary and lifestyle factors [18]. In elderly individuals, a notable shift occurs towards reduced microbial diversity, which is influenced by age‐related changes in diet, lifestyle, and overall health [19]. This pattern of low‐high‐low gut microbiota diversity throughout life mirrors that of low‐high‐low vaccine immunogenicity. Indeed, evidence suggests that many of the factors that are reported to influence vaccine immunogenicity do so, at least in part, by modulating the composition of the microbiota. In this review, we synthetise the evidence from human, preclinical and experimental studies which indicate that the microbiota is a key factor regulating immune responses to vaccination, discuss the potential mechanisms involved, and provide our thoughts on future directions for the field.

## The gut microbiota is central to immune homeostasis and impacts vaccine immunogenicity

There is now considerable evidence that the gut microbiota has a broad role in regulating the function of both the innate [20, 21, 22, 23, 24, 25] and adaptive [26, 27, 28, 29] arms of the immune system. Dysregulation of this microbiota‐immune axis is associated with increased risk of developing disorders such as asthma, arthritis and inflammatory bowel disease [30, 31], and has been shown to strongly influence the efficacy of cancer immunotherapies [32, 33]. The impact of the gut microbiota is particularly evident in germ‐free animals, which are devoid of a microbiota. Germ‐free animals, for example, have altered physiology and immune function compared to their conventionally colonised counterparts including smaller lymph nodes, altered gut‐associated lymphoid structures (GALT), increased susceptibility to infection, diminished T helper 17 (Th17) cell populations, and fewer IgA‐producing plasma cells [34, 35, 36, 37, 38, 39, 40, 41]. Given the substantive evidence that the gut microbiota plays a critical role in regulating immune development, it is perhaps unsurprising that the gut microbiota can also impact vaccine immunogenicity.

### Evidence from clinical studies that the microbiota impacts vaccine immunogenicity

Over the past 20 years, there have been a plethora of clinical studies investigating whether there is a relationship between the composition of the gut microbiota and vaccine immunogenicity. The majority of these studies have been observational cohort studies conducted in infants (< 2 years of age) that focused on uncovering associations between the composition of the human gut microbiota and vaccine immunogenicity [42, 43, 44, 45, 46, 47, 48, 49, 50, 51, 52, 53, 54, 55, 56, 57, 58, 59, 60, 61, 62, 63, 64] (Table 1). To date, most of these studies have used relatively low‐resolution microbiota profiling technologies, such as 16S rRNA gene sequencing or phylogenetic microarrays, limiting the detection of species‐ or strain‐specific associations.

**Table 1:** Association studies linking the human gut microbiota to vaccine immunogenicity. ↑, increased number of; ↓, decreased number of; BCG, Bacille Calmette‐Géurin; Bif, Bifidobacterium; IPV, inactivated poliovirus; NEG, negatively; OPV, oral polio virus; PCV, Pneumococcal conjugate vaccine; POS, positively.

References	Age	Sample size	Vaccines	Taxa associations reported	Country
[42]	Infants (6–15 weeks)	*n* = 48	BCG	↑ Actinobaceria & Bif POS associated with T cell responses. ↑ Pseudomondales & Enterobacteriales NEG associated with T cell reponses.	Bangladesh
			OPV	↑ Bif POS correlated with B cell responses. ↑ Actinobaceria POS associated with T & B cell responses. ↑ Coriobacteriaceae POS associated with T cell responses. ↑ Bif, *B. longum* & *B. longum* subsepcies *infantis* POS associated with T cell responses. ↑ Pseudomondales NEG associated with B & T cell responses	
			Hep B	↑ Actinobacteria & *Rothia* POS associated with B cell responses. ↑ Pseudomondales NEG associated with T cell reponses. ↑ High diversity NEG associated with T cell responses.	
			Tetanus Toxoid	↑ Actinobaceria POS associated with antigen specific T cell responses. ↑ *B. longum* subspecies *infantis* POS correlated with antigen specific T cell responses. ↑ Actinomycetales POS associated with antigen specific B cell reponses. ↑ Pseudomondales & Enterobacteriales NEG associated with antigen specific T cell responses. ↑ High diversity was NEG associated with antigen specific T cell responses.	
[43]	Infants (6–18 weeks)	*n* = 117	Rotarix®	↑ Bacilli POS correlated with vaccine response. ↑ Bacteriodetes NEG correlated with vaccine response.	Ghana
[44]	Infants (6 weeks)	*n* = 16	Rotarix®	↑ Ratio of Gram negative over Gram positive bacteria POS correlated with B cell responses. ↑ Firmicutes, Proteobacteria, *Serratia* & *E. coli* POS associated with B cell responses.	Pakistan
[45]	Infants (6–10 weeks)	*n* = 170	Rotarix®	No consistent differences between responders & non‐responders in composition or diversity of the 16S bacterial microbiota.	India
[46]	Infants (6 weeks & 2 years)	*n* = 280, *n* = 249	BCG	↑ Bif POS associated with antigen specific T cell responses.	Bangladesh
			OPV	↑ Bif POS associated with antigen specific B cell responses.	
			Hep B	↑ Bif POS associated with T cell responses.	
			Tetanus Toxoid	↑ Bif POS associated with antigen specific T & B cell responses.	
[47]	Infants (2 months)	*n* = 45	RotaTeq®	↓ Proteobacteria & Enterobacteriaceae was POS associated with B cell responses. ↑ *A. muciniphilia*, *B. fragilis*, *Lactobacillus* spp., *B. longum* & *B. bifidum* NEG associated with B cell responses. ↑ Fusobacteria & *Eggerthella* was POS associated with B cell responses.	Nicaragua
[48]	Infants (6–15 weeks)	*n* = 122	Rotarix®	↑ *Streptococcus* & Enterobacteriaceae POS associated with antigen specific B cell responses. ↑ *E. lenta* POS correlated with antigen specific B cell responses. ↑ *Bocaparvovirus* POS correlated with B cell response. ↑ *Enterovirus* B, *Cosavirus* A & phage richness NEG associated with antigen specific B cell responses.	Ghana
[49]	Infants (12–18 months)	*n* = 101	PCV10	Vaginal birth POS associated with antigen specific B cell responses. ↑ Bif & *E. coli* POS associated with antigen specific B cell responses.	Denmark
	Infants (12–18 months)	*n* = 66	MenC	Vaginal birth POS associated with antigen specific B cell responses. ↑ *E. coli* POS associated with antigen specific B cell responses	
[58]	Adults	*n* = 21	RV144	Baseline composition of the gut microbiota associated with vaccine response.	Switzerland
[59]	Infants (3 weeks to 2 years)	*n* = 101	DTaP‐Hib	Antibiotic use NEG associated with vaccine response.	USA
			PCV13		
[61]	Adults	*n* = 17	Ty21a	↑ Clostridiales POS associated with T cell responses. ↑ Microbial diversity POS associated with T cell responses	USA
[62]	Infants (2 months)	*n* = 107	IPV & OPV	↑ Breast feeding POS associated with B cell responses. ↑ Firmicutes & Clostridia were NEG associated with B cell responses. ↓ Actinobacteria NEG associated with B cell responses.	China
[63]	Infants (6–11 months)	*n* = 704	OPV	↑ Non‐polio enteroviruses NEG associated with B cell responses	India
[64]	Infants (6 weeks–12 months)	*n* = 83	PCV13	↑ *S. oralis* NEG associated B cell response.	USA
			Tetanus Toxoid	↑ *S. aureus*, *E. coli*, *S. thermophilus* & *A. vaginalis* POS associated with antigen specific B cell responses. ↑ Beta diversity NEG associated with antigen specific B cell response. ↑ *A. aeriphila* NEG associated with antigen specific B cell response.	
[65]	Infants (1–7 weeks)	*n* = 66	BCG (*M. tuberculosis*)	↑ *B. longum* subsp. *infantis* POS associated with T cell responses. ↑ *B. thetaiotaomicron* NEG associated with T cell responses.	South Africa
[69]	Infants (0–4 months)	*n* = 20	IPV	↑ Bif POS associated with antigen specific B cell responses. ↑ *B. longum‐infantis* POS associated with antigen specific B cell responses.	France
[70]	Infants (6–24 months)	*n* = 560	DTaP	Antibiotics NEG associated with antigen specific B cell responses levels	USA
			IPV		
			Hib		
			PCV		
[75]	Infants (1–6 weeks)	*n* = 486	Rotarix®	↑ Microbiota diversity NEG correlated with ORV immunogenicity.	Malawi, India, UK
[78]	Adults	*n* = 63	Rotarix®	Vancomycin administration did not impact overall vaccine response.	Netherlands
[79]	Infants (6–11 months)	*n* = 754	OPV	Azithromycin administration did not improve vaccine immunogenicity.	India
[81]	Infants (6–52 weeks)	*n* = 339	OPV	↑ *Campylobacter* and enteroviruses NEG associated with B cell responses.	Bangladesh
			Rotarix®	↑ Enteroviruses NEG associated with B cell responses.	
[82]	Adults	*n* = 33	Fluzone	Antibiotic use NEG associated with antibody response.	USA

Although no single bacterial phylum, genus, species or strain has been identified to be associated with enhanced vaccine immunogenicity across all studies, the relative abundance of phylum Actinomycetota (formerly Actinobacteria), which predominantly consists of the genus *Bifidobacterium* in the infant gut microbiota, has been reported to be positively correlated with vaccine immunogenicity in a number of different studies [42, 46, 49, 50, 62, 65]. *Bifidobacterium* spp. are the dominant constituent of the infant gut microbiota in humans [15, 66, 67], due in large part by their ability to efficiently use human milk oligosaccharides (HMOs) in breastmilk as an energy source [68]. In support of a causal relationship between *Bifidobacterium* and enhanced vaccine responses, a clinical trial randomising infants to receive a specialised infant formula containing bifidogenic factors was reported to increase anti‐polio IgA responses after vaccination with a parenterally administered pentavalent vaccine [69]. Furthermore, antibiotic exposure, formula feeding, and C‐section delivery, which are all factors reported to decrease *Bifidobacterium* levels in the developing gut microbiota [15, 66], are also factors that are negatively associated with vaccine immunogenicity [49, 59, 70]. Exactly how *Bifidobacterium* spp. may lead to enhanced responses to vaccination remains to be shown, however, administering *Bifidobacterium* to infants can modulate immune function in early life in multiple different ways that would likely have a beneficial effect on vaccine responses [69, 71, 72]. For example, administration of *Bifidobacterium infantis* EVC001 to infants was shown to lower enteric inflammation and pro‐inflammatory cytokine production [71, 72]. *Bifidobacterium* produced metabolites, such as indole‐3‐lactic acid and short‐chain fatty acids (SCFAs), have also been shown to have a range of anti‐inflammatory properties including promoting the production of secretory IgA by B cells in the gastrointestinal tract [73] and the generation of specialised subsets of regulatory B cells, which are important for suppressing inflammation [74]. Together these data support a particularly important role for *Bifidobacterium* strains in promoting optimal immune development in early life including the mediation of optimal responses to vaccination.

### The microbiota and responses to oral vaccines

The relationship between the composition of the infant gut microbiota and immune responses to the oral rotavirus vaccine (ORV) has been particularly well studied [42, 43, 44, 45, 46, 47, 48, 75]. This increased attention may be in part due to the oral route of administration for ORV, which increases the likelihood of direct interactions with the gut microbiota. Additionally, it has been postulated in several previous studies that the lower efficacy of ORV in LMICs may be mediated in part by differences in the composition of the gut microbiota in LMICs compared to HICs [76, 77]. To date, these studies have not, however, revealed a definitive relationship between the composition of the gut microbiota and ORV immunogenicity. Rather they have reported conflicting results. Certain bacterial taxa such as *Enterobacteriaceae* have been reported to be both positively [48] and negatively [47] correlated with rotavirus‐specific IgA antibodies in serum in different cohort studies. Other studies still have found no significant associations [45]. Furthermore, a recent randomised controlled trial (RCT) that evaluated the impact of antibiotics on the immunogenicity of ORV in adults found that while there was increased faecal shedding of rotavirus following antibiotics, there was no difference in immunogenicity [78]. Similarly, Grassly *et al*. [79] reported no effect of azithromycin on the immunogenicity of the oral poliovirus vaccine in infants (6–11 months old), despite the fact that other studies have shown that an increased relative abundance of Actinomycetota species is positively associated with vaccine responses in infants [42, 46, 62]. There are several possible explanations for the inconsistency between these studies including differences in pre‐existing immunity, environmental exposure and other enteric infections. It is also possible that researchers have been assessing associations with the wrong component of the microbiome, as a recent study has reported that gut virome diversity and the presence of certain viruses (Enterovirus B, Cosavirus A) were negatively associated with ORV seroconversion [48]. The virome is a comparatively understudied component of the gut microbiome but can directly contribute to host immune variation and shape bacterial populations [80]. Enterovirus abundance has also been shown to be negatively associated with vaccine responses to both the oral poliovirus and rotavirus vaccines in Bangladeshi infants [81] and the oral polio virus vaccine in Indian infants [63]. A single study to date has evaluated the relationship between the gut microbiota and the orally administered live‐attenuated Ty21a vaccine for the prevention of typhoid fever. The authors of this study found no association between the composition of the microbiota and the magnitude of IgG and IgA responses in serum induced following administration of this vaccine. However, they were able to evaluate associations with the kinetics of the response and reported that lower gut microbiota diversity was associated with late vaccine responders [61]. The authors also reported that the administration of the Ty21a vaccine did not alter the composition or diversity of the gut microbiota.

### Antibiotic exposure and responses to parenteral vaccines

Although the studies outlined above suggest that there may not be a negative impact of antibiotics on immune responses to oral vaccines, several recent studies suggest that antibiotic exposure prior to immunisation may have a deleterious effect on parenteral (non‐oral) vaccine immunogenicity [70, 78, 79, 82]. Hagan *et al*. [82], for example, conducted two RCTs investigating the impact of a cocktail of neomycin, vancomycin, and metronidazole on strain‐specific antibody responses in adults immunised subsequently with an influenza vaccine. Although the sample size in this study was modest, they also performed comprehensive systems immunology assessments including profiling of the blood transcriptome and metabolome as well as the composition of the faecal microbiota. The authors reported that antibiotics negatively impacted influenza vaccine immunogenicity, but only for individuals with low antibody titres pre‐vaccination. Further supporting a negative impact of antibiotics on parenteral vaccine immunogenicity, a retrospective analysis of 560 children under the age of two in the USA found that prior antibiotic treatment was associated with lower antibody titres against the diphtheria‐tetanus‐acellular pertussis (DTaP), inactivated polio (IPV), *Haemophilus influenzae* type b (Hib), and pneumococcal conjugate (PCV) vaccines [70]. In contrast, however, another study did not find an association between antibody responses to infant immunisations and antibiotic exposure [83], although there was a modest sample size of antibiotic exposed infants and the study was not specifically designed to address this link. It is also important to consider that the impact of antibiotics on the composition of the gut microbiota is highly individual specific depending on the pre‐treatment composition [17]. Perhaps more importantly, Hagan *et al*. found an impact of antibiotic treatment only in participants with low pre‐existing immunity, which was not specifically assessed in any of the studies that found no association between antibiotic exposure and vaccine immunogenicity. Taken together, these data indicate that antibiotic exposure can modulate the immunogenicity of at least some vaccines in humans, particularly in cases where there is low or no pre‐existing immunity.

### Probiotics and responses to vaccination

The impact of administering probiotics on vaccine immunogenicity in humans has been assessed in several recent systematic reviews [84, 85]. Consistent with a lack of an association between antibiotic exposure and oral vaccine immunogenicity, a systematic review concluded that there was no evidence of a beneficial effect of probiotics on seroconversion following oral vaccine administration [86]. Data, however, suggest that there may be an impact of probiotics on parenteral vaccine immunogenicity. For example, a systematic review of the data from 623 adults across nine RCTs concluded that pre‐ or probiotics before influenza vaccination led to higher rates of seroconversion against the H1N1, H3N2, and B strains of influenza [84]. The probiotics used in these studies were predominantly *Lactobacillus* spp. (*Lactobacillus casei* was the most widely used) whereas the prebiotics were largely fructo‐oligosaccharides. Zimmermann and Curtis [85] subsequently conducted a more wide‐ranging systematic review of data from 26 RCTs (3812 participants) assessing the effect of probiotics on the immunogenicity of 17 different vaccines. They reported that there is evidence supporting a beneficial effect of probiotics on vaccine immunogenicity, although only half of studies reported a significant effect. The most consistent outcome identified was increased responses to parenteral influenza vaccination in adults (*n* = 6 studies), although the authors noted that given the considerable amount of variation between studies (e.g. 40 different probiotic strains were assessed across the 26 studies) future work establishing which are the optimal probiotic strains to use and the optimal timing of their administration is needed [85].

## The role of the microbiota in regulating responses to COVID‐19 vaccines

Several human cohort studies have assessed potential associations between the composition of the gut microbiota and the immunogenicity of COVID‐19 vaccines, particularly responses to the Pfizer/BioNTech BNT162b2 mRNA vaccine (Table 2) [50, 51, 52, 53, 54, 55, 56, 57]. Although these studies all reported significant associations between the gut microbiota and COVID‐19 vaccine immunogenicity, no taxa or genera in the microbiota were consistently found to be associated with vaccine immunogenicity across studies. Possible explanations for this lack of consistency between studies is the fact that the different studies to date were conducted in different regions (China, USA, UK, Hong Kong, Japan, Canada), and used a mix of microbiota profiling technologies (either shotgun metagenomics or 16S rRNA gene sequencing). Taxa reported to be associated with increased COVID‐19 vaccine immunogenicity include *Bifidobacterium* [50, 51], *Bilophila* [53, 55], *Roseburia* [51, 57], and *Alistipes* [50, 56]. *Bifidobacterium* is particularly interesting in this context given its numerous prior associations with vaccine immunogenicity in infants [42, 46, 49, 50, 62]. However, although *Bifidobacterium* is predominant in the infant gut microbiota, it is typically detected less often and at a lower relative abundance in adults [87]. Supporting a link between the composition of the microbiota and responses to COVID‐19 vaccines, two recent RCTs have reported that supplementation with *Lactobacilli* probiotics leads to higher antibody titres following either infection with, or vaccination against, SARS‐CoV‐2 [88, 89]. Furthermore, a recent retrospective analysis reported that individuals exposed to antibiotics within a 6‐month period prior to receiving the BNT162b2 vaccine had lower seroconversion rates after the first, but not the second dose [90]. These data reinforce the suggestion that the impact of the gut microbiota may be most pronounced in those without pre‐existing immunity [82]. Given all these data, we were surprised to find that germ‐free or antibiotic exposed mice exhibited antigen‐specific B and T cell responses to the BNT162b2 vaccine on par with conventionally colonised mice [91]. Given that mRNA vaccines are typically more immunogenic than other vaccine platforms [92, 93, 94], this may explain why they are less influenced by signals from the gut microbiota in animal models. Taken together, these data suggest that further research is needed to understand what role the microbiota plays in COVID‐19 vaccine immunogenicity.

**Table 2:** Association studies linking gut microbiota to COVID‐19 vaccine immunogenicity. ↑, increased number of; ↓, decreased number of; Bif, Bifidobacterium; NEG, negatively; POS, positively.

References	Age	Sample size	Vaccine	Taxa associations reported	Country
[50]	Adults (18–67 years)	*n* = 37	CoronaVac	↑ *B. adolescentis* POS associated with B cell responses. ↑ *B. vulgatus*, *B. thetaiotaomicron* & *R. gnavus* NEG associated with B cell responses.	Hong Kong SAR
		*n* = 101	BNT162b2	↑ *E. rectale*, *R. faecis*, *B. thetaiotaomicron* & *B. sp*. OM05‐12 POS associated with B cell responses.	
[51]	Adults	*n* = 40	CoronaVac	↑ *P. dorei*, *B. massiliensis* & *D. formicigenerans* POS associated with antigen specific B cell responses. ↑ *F. prausnitzii* NEG associated with antigen specific B cell responses.	Hong Kong SAR
		*n* = 121	BNT162b2	↑ *B. adolescentis*, *B. bifidum* & *R. faecis* POS associated with antigen specific B cell responses.	
[52]	Adults	*n* = 207	BBIBP‐CorV	↑ *C. aerofaciesn*, *F. saccharivorans*, *E. ramulus* & *V. dispar* POS associated with B cell responses. ↑ *L. asaccharolyticus* was NEG associated with B cell responses	China
[53]	Adults	*n* = 15	ChAdOx1	↓Gut microbiota diversity NEG associated with vaccine response. ↑ *Bilophila* POS associated with vaccine response. ↑ *Streptococcus* NEG associated with vaccine response.	UK
		*n* = 28	BNT162b2		
[54]	Adults (Pregnant women)	*n* = 97	CoronaVac	↑ *Anaeromyces*, *B. plebeius*, *C. tanakaei*, *Acatinomyces*, *P. distasonis*, *Coprobacillus*, *Anaeromasilibacillus*, *B. longum*, *E. ramosum*, *S. parasanguinis*, *S. salivarius* & *Ruminococcus* spp. POS associated with antigen specific B cell responses	China
			BNT162b2		
[55]	Adults	*n* = 16	BNT162b2	↑ Desulfobacterota & *Bilophila* POS correlated with antigen specific B cell responses. *↑ Bacteroides* NEG associated with antigen specific B cell responses.	USA
			Spikevax		
[56]	Adults	*n* = 95	BNT162b2	Microbial diversity NOT associated with adaptive vaccine response.	Japan
[57]	Adults	*n* = 52	BNT162b2	↑ *E. rectale*, *R. faecis*, *B. thetaiotaomicron*, *Bacteroides* sp. *Prevotella*, *Haemophilus*, *Veillonella & R. gnavus* OM05‐12 POS associated with antigen specific B cell responses. ↑ *B. bifidum* & *A. intestini* NEG associated with antigen specific IgG antibody avidity. ↑ *B. animalis*, *B. plebeius* & *B. ovatus* POS associated with anitigen specific IgG antibody avidity	Canada
[60]	Adults	*n* = 30	Sinovac	↑ *C. leptum*, *L. ruminis*, *R. toques* POS correlated to antigen specific B cell responses. ↑ *P. copri* NEG associated with antigen specific B cell responses.	China

## Other evidence from pre‐clinical studies that the microbiota impacts vaccine immunogenicity

As discussed above, clinical studies conducted to date have in many, but not all, cases supported the hypothesis that a healthy gut microbiota plays an important role in mediating optimal immune responses to vaccination. Establishing causal relationships and determining the mechanisms involved, however, is usually more tractable in pre‐clinical models. The majority of the pre‐clinical studies conducted in this field to date have been conducted in mice [91, 95, 96, 97, 98, 99, 100, 101, 102], although the impact of the microbiota on vaccine responses has also been investigated in a range of other species including chickens [103, 104, 105], pigs [106, 107, 108, 109, 110, 111, 112, 113, 114, 115], Syrian hamsters [116, 117] and non‐human primates [101, 118, 119]. The vaccines investigated range from live‐attenuated to inactivated and subunit vaccines and the gut microbiota has been shown to impact immune responses to many different types of vaccine in these studies.

Antibiotics have been used extensively in pre‐clinical models to investigate the relationship between the gut microbiota and vaccine responses. These studies have used a range of broad and narrow spectrum antibiotics to either substantially deplete the bacterial component of the gut microbiota or to deplete specific bacteria [91, 95, 96, 97, 98, 99, 100, 101, 102, 105]. For example, mice exposed to a cocktail of neomycin and ampicillin from *in utero* to the time of weaning, were found to have significantly impaired antigen‐specific total IgG responses up to 12‐weeks post vaccination with a range of licenced infant vaccines including the live‐attenuated BCG vaccine and the adjuvanted PCV13, meningococcal serogroup B and C, and hexavalent Infanrix Hexa vaccines [98]. Interestingly, adult mice exposed to the same antibiotic cocktail did not show impaired antibody responses to the BCG or PCV13 vaccines [98], suggesting that the gut microbiota may play a more significant role in modulating vaccine responses in early life. Restoration of the gut microbiota post antibiotic treatment through faecal microbiota transfer (FMT) restored impaired vaccine responses to the PCV13 vaccine [98]. Antibiotic‐treated chickens that had their microbiota reconstituted by FMT were similarly shown to have higher haemagglutination titres following vaccination with avian influenza (H9N2) vaccine, compared to non‐reconstituted chickens [105]. Similarly, impaired vaccine responses to the model antigen ovalbumin (OVA) in mice and rhesus macaques after vancomycin administration were rescued when sufficient time was allowed for the restoration of microbial diversity prior to vaccination [101].

In addition to antibiotic exposure, germ‐free animals have also been used to investigate the relationship between the gut microbiota and vaccine responses [91, 95, 96, 100, 102, 105]. Germ‐free mice have significantly impaired antibody responses to OVA [96] as well as impaired antibody and T cell responses to adjuvanted model antigen human serum albumin (HSA) [95]. Interestingly, given the inconsistent data in human studies assessing links between the microbiota and oral vaccine responses, germ‐free mice orally vaccinated with ORV had higher levels of antigen‐specific IgG and IgA compared to their conventional counterparts [102]. Antibiotic‐treated mice similarly exhibited enhanced serum IgG and IgA post oral administration of a murine adapted rotavirus [102]. Germ‐free pigs have also been used to study the impact of the gut microbiota and/or probiotics on responses to ORV [106, 107, 108, 109, 110, 111, 112, 113, 114, 115]. For example, neonatal germ‐free pigs co‐colonised with *Lactobacillus rhamnosus* GG and *Bifidobacterium animalis lactis* Bb12, followed by ORV vaccination, showed significantly enhanced ORV immunogenicity and reduced susceptibility to infection [106, 108].

## Potential mechanisms underlying the gut microbiota's impact on vaccine immunogenicity

**Fig. 1:**
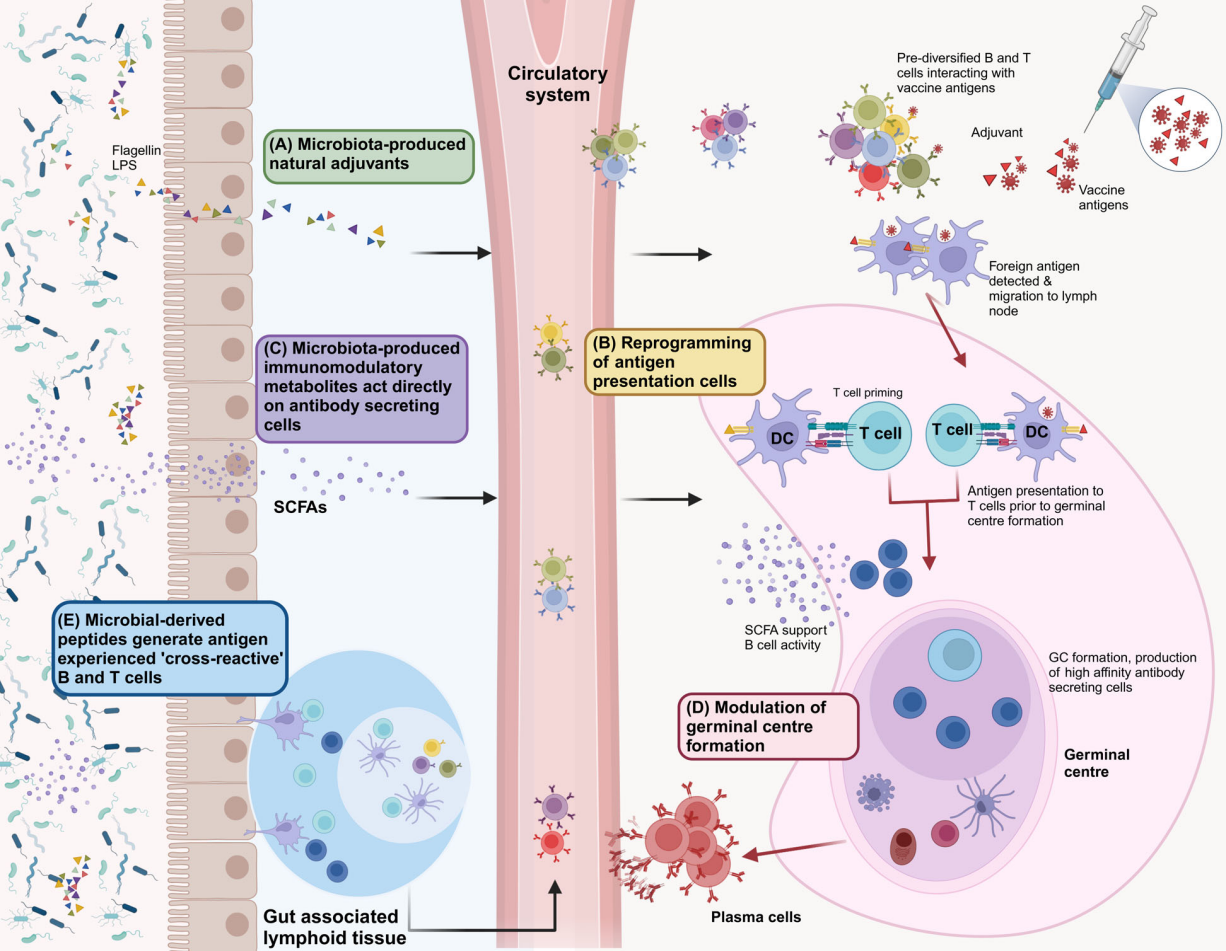
Potential mechanisms underlying the gut microbiota's impact on vaccine immunogenicity. (A) Flagellin and LPS (lipopolysaccharide) produced by the gut microbiota can potentially act as natural adjuvants by (B) activating pattern recognition receptor (PRR) pathways on antigen presenting cells such as DCs. (C) Microbiota‐produced immunomodulatory metabolites such as short chain fatty acids (SCFAs) can act as an energy source for B cells and support antibody production. (D) Signals from the microbiota have been shown to impact germinal centre (GC) formation through an unknown mechanism. GC formation is crucial for the production of high‐affinity antibodies post‐vaccination. (E) B and T cells directed against gut microbiota‐encoded antigens can cross‐react with vaccine epitopes and can positively or negatively impact vaccine responses. Figure was generated using Biorender.com.

Given the wide‐ranging mechanisms through which the gut microbiota can influence immune function in other contexts [20, 21, 22, 23, 24, 25, 26, 27, 28, 29], there are numerous plausible ways that the microbiota could modulate vaccine immunogenicity. Interestingly, despite intensive research, the mechanisms involved remain incompletely understood, likely reflecting the fact that the microbiota can potentially influence many of the key steps in the innate and adaptive responses needed for optimal vaccine immunogenicity (Fig. 1). In the next part of this review, we discuss the evidence for each of the different mechanisms that have been proposed to date.

### Natural adjuvant hypothesis

Many non‐live vaccines require formulation with an adjuvant, such as alum, to promote strong immune responses to vaccination by activating pattern recognition receptor (PRR) pathways in antigen presenting cells. Increasing evidence suggests that immunomodulatory products such as lipopolysaccharide (LPS), flagellin, peptidoglycan and microbial DNA produced by the gut microbiota can act as a reservoir of natural adjuvants which can assist adaptive immune responses [120]. These products can cross the gut epithelial barrier into the circulatory system where they are sensed via similar pathways as those involved in activating the immune response to vaccine adjuvants. For example, mice deficient in Toll‐like receptor (TLR) 5, the PRR that recognises the microbial product flagellin, have been shown to have significantly impaired antibody responses to a non‐adjuvanted influenza vaccine [100]. Furthermore, administering a flagellated, but not an aflagellated, strain of *Escherichia coli* to germ‐free mice via oral gavage restored antibody responses to an influenza vaccine. Other studies have similarly shown that fusing bacterial flagellin to the influenza protein haemagglutinin elicits stronger immune responses compared to commercially available influenza vaccines [121, 122]. Innate‐like B‐1 cell antibody responses to Group A *Streptococcus* have also been found to be dependent on an intact gut microbiota and on MyD88, a key adaptor protein downstream of multiple TLRs [123]. Nucleotide oligomerisation domain (NOD)‐like receptors are another type of PRR required for sensing bacterial cell wall components as well as bacterial toxins. The impaired antibody response in germ‐free mice following intranasal immunisation with HSA and cholera toxin was restored by administration of muramyl dipeptide (which is recognised by NOD2) or by colonisation with bacteria that activate the NOD2 pathway [95]. Further work is needed to understand whether sensing of microbiota produced products by other PRRs also play a role in mediating the influence of the microbiota on vaccine responses.

### Reprogramming of antigen presentation cells

Dendritic cells (DCs) play an important role in presenting vaccine antigens to T and B cells, in a process that is dependent on PRR signalling resulting in DC activation [4]. Evidence is emerging that the microbiota can reprogram these antigen presenting cells thereby modulating vaccine responses. A study by Ruane *et al*. demonstrated that after intranasal vaccination with inactive cholera toxin, TLR‐mediated sensing of the microbiota by lung DCs led to IgA^+^ B cells upregulating expression of α4β7 integrin and CC‐chemokine receptor 9 (which are important for migration of these B cells to the gut [124]), and protection against subsequent challenge. Germ‐free mice who received intranasal vaccination with inactive cholera toxin had a significant reduction in the levels of antigen‐specific IgA. Additionally, it has also been shown that the gut microbiota regulates the production of type I interferons (IFN‐I), pivotal cytokines that mediate antiviral responses, by a specialised subset of DCs called plasmacytoid DCs (pDCs) [125]. IFN‐I production by pDCs in turn led to an epigenetic reprogramming of conventional DCs, posing them for enhanced responses [125]. Macrophages, a critical cell of the innate immune system, are also involved in antigen presentation and are reportedly influenced by the gut microbiota [100, 126]. For example, macrophages isolated from antibiotic‐treated mice were shown to have decreased expression of genes associated with antiviral immunity, defective responses to type I and type II interferons as well as impaired capacity to limit viral replication [126]. Moreover, sensing of microbiota produced flagellin by macrophages, but not DCs, was shown to be vital for antibody responses to non‐adjuvanted influenza vaccine in mice, as mice depleted of macrophages failed to mount antigen‐specific antibody responses, whilst the absence of functional DCs had no impact [100]. Microbiota‐derived metabolites have also been shown to modulate antigen presentation (see below) [127].

### Microbiota‐derived metabolites

Another mechanism through which the microbiota may influence immune responses to vaccination is via the production of immunomodulatory metabolites that enter the circulatory system. Short chain fatty acids (SCFAs), for example, are produced through the fermentation of complex fibres [128]. SCFAs produced by the gut microbiota are primarily butyrate, propionate and acetate [129]. SCFAs have been shown to support the increased energy demands of antibody production by B cells, by increasing acetyl‐CoA and regulating metabolic sensors to increase oxidative phosphorylation, glycolysis, and fatty acid synthesis [130]. A subsequent study, however, showed that SCFAs led instead to reduced antibody responses to intragastrically administered OVA, which was mediated by epigenetic modifications of the *AICDA* and *PRDM1* genes in B cells, negatively impacting somatic hypermutation and class‐switching [131]. Further work needs to be done to address the conflicting results around the effect that SCFAs can have on B cells and the subsequent potential impact on vaccine responses. In an *in vitro* model, butyrate and propionate have been shown to significantly reduce the expression of IL‐6, a critically important cytokine for mounting immune responses, by human monocyte derived DCs [132]. Moreover, both butyrate and propionate have been shown to significantly impair antigen presentation by mature DCs to CD8^+^ T cells by inhibiting the expression of co‐stimulatory molecules such as CD80 and CD40 as well as the production of IL‐12 [127]. Tryptophan metabolites and secondary bile acids have also been shown to have immunomodulatory effects [133, 134]. Tryptophan metabolites, for example, have been shown to signal through the aryl hydrocarbon receptor (AhR) to regulate the differentiation and function of several immune cells that play key roles in immune responses to vaccination including macrophages, DCs, T cells and B cells [135].

### Modulation of germinal centre formation

During infection or vaccination, specialised microstructures called germinal centres (GCs) develop in the B cell follicles of secondary lymphoid organs, such as lymph nodes and the spleen. B cell activation occurs outside the GC, whereby binding with cognate CD4^+^ T helper cells facilitates entry through expression of survival and co‐stimulatory signals [136]. GCs support the maturation and differentiation of B cells which ultimately leads to the production of high‐affinity antibodies [137]. Modification of the formation or function of GCs could in principle be responsible for alterations associated with B‐cell‐dependent immune responses. For example, the presence of *Bacteroides acidifaciens* within the gastrointestinal tract has been shown to promote the formation of GCs in gnotobiotic mice [138], which in principle could lead to increased antibody production and more robust responses to vaccination. Studies have also demonstrated that mice sourced from different vendors exhibit distinct gut microbiota compositions, which can significantly affect their immune response to *Plasmodium yoelii* (a parasite responsible for malaria) infection including the number of GC B cells in the spleen, and the levels of parasite‐specific antibodies induced by infection [139, 140]. Interestingly, mice treated with vancomycin either the day of the primary parasite infection or 7 days after primary parasite infection showed increased levels of parasite‐specific IgG, T follicular helper (Tfh) cells and GC B cells compared to non‐vancomycin‐treated infected mice, however, blocking GC formation resulted in increased parasite burden regardless of vancomycin treatment [140]. Collectively, these data suggest that signals from the gut microbiota to GCs were required to control parasite infection in mice, however, identifying the precise mechanisms involved requires further investigation. Conversely, GC formation can be disrupted by exposure to LPS [141], perhaps suggesting that a shift towards a gram‐negative dominated microbiota could hinder GC formation following vaccination. Supporting this hypothesis, antibiotic exposure has been shown to lead to increased abundance of gram‐negative Pseudomonadota [142] and inflammation [143].

### Cross‐reactive B and T cells produced by the microbiota

A growing body of evidence suggests that similarity between epitopes expressed by the microbiota and vaccine/pathogen epitopes can lead to presence of cross‐reactive B or T cells prior to vaccination or infection [144, 145, 146]. For example, T‐independent immune responses to the Pneumovax vaccine (a subunit vaccine administered to children and adults offering protection against pneumococcal disease) have been shown to largely involve pre‐diversified marginal zone and related B cell subsets that recognise a broad range of commensal bacterial species [147]. Furthermore, early on in the COVID‐19 pandemic, SARS‐CoV‐2 naïve individuals were found to have pre‐existing B cell immunity to the virus [148, 149, 150]. Subsequent evidence indicated that this was only partially driven by exposure to other coronaviruses [151, 152] and instead, antigens derived from commensals including *Streptococcus*, *Bifidobacterium* and *Bacteroides* led to the production of cross‐reactive antibodies [153, 154]. Pre‐existing antibodies against the SARS‐CoV‐2 Spike protein was observed in human serum samples collected in 2016 and 2020, as well as in naïve SPF mice [153]. Monoclonal antibodies (mAbs) that bound the Spike protein were isolated from these SPF mice and were subsequently shown to bind to whole cell lysates obtained from the commensal gut microbiota of both humans and mice. The presence of the oral commensal *Streptococcus salivarius* also been shown to lead to the generation of cross‐reactive anti‐Spike antibodies [154] and supplementation of the probiotic *S. salivarius* K12 induced significantly increased anti‐Spike salivary IgG and IgA responses as well as an increased reactivity towards the receptor binding domain (RBD) of the Omicron/B1.1.529 variant of SARS‐CoV‐2 in humans vaccinated with BNT162b2 mRNA vaccine [154]. In contrast, microbiota‐derived antibodies that cross‐react with gp41, an antigen in a candidate human immunodeficiency virus (HIV) vaccine, diverted the B cell response to this candidate vaccine from neutralising to non‐neutralising [155]. Together these data suggest that the microbiota is capable of priming B and T cells which cross‐react with vaccine antigens, and that these effects can either support or impair vaccine responses depending on the context.

## Ecological interactions between the gut microbiome and live‐attenuated vaccines

In the complex ecosystem of the gut microbiome, direct microbial interactions and competition among microorganisms are pivotal in shaping dynamics of the overall system, and in principle, may impact the immunogenicity of orally administered live‐attenuated vaccines. To date, the impact of direct interactions between the microbiome and the immunogenicity of live‐attenuated oral vaccines is largely unexplored. However, direct competitive interactions are well‐documented in other contexts [156], particularly for bacteria. Microbial species compete for resources such as dietary polysaccharides and host‐derived glycans, with more metabolically efficient species outcompeting slower metabolisers [157]. This is a significant factor in shaping the overall microbiota composition and metabolic output. In the context of an orally administered live‐attenuated vaccine, the availability of specific nutrients and competition from other species may influence persistence in the gut and subsequent immune interactions. Microbial species may also have antagonistic interactions with each other through the production of targeted antimicrobial compounds such as bacteriocins—ribosomally synthesised peptides that inhibit closely related species that may be competing for similar resources [158]. Conversely, synergistic interactions could enhance vaccine efficacy, for instance, through cross‐feeding where existing microbiota species produce compounds that support the growth of live‐attenuated vaccine strains [159]. The human microbiome also contains a vast viral component, including many eukaryotic viruses present asymptomatically [160] which could drive competitive virus‐virus interactions. Modelling of population level infection data suggests virus–virus interactions exist and can shape infection outcomes although virtually nothing is known of this in the context of vaccine responses [161].

## Future perspectives

The literature reviewed here provides strong evidence that the gut microbiota, particularly the bacterial component, can impact responses to vaccination in humans, with varying effects depending on the vaccine technology platform, antigen, age of the individual and route of administration. This not only points to a cause for sub‐optimal vaccine immunogenicity in some individuals or populations, but excitingly identifies potential safe and tractable approaches to enhance protection offered by current licenced vaccines. Given the large body of correlation‐based evidence in humans, we propose that establishing a causal role for specific members of the microbiota in modulating vaccine immunogenicity is now of the utmost importance.

Correlation‐based assessments, such as those used in the cohort studies discussed above, are limited in this regard, particularly given that issues related to compositional data (such as taxa relative abundances) are known to lead to spurious associations in some cases. A standard approach often used in other contexts is to identify associations in large cohorts and then validate causality using *in vivo* or *in vitro* models. Advances in molecular profiling technologies will without doubt aid this process, particularly for *in vivo* models. For example, spatial transcriptomics technologies can enable the investigation of defects in GC formation with unprecedented resolution. Similarly, single cell multi‐omics allows for simultaneously identifying changes in frequency, transcriptome, and epigenome of individual immune cells. Pairing these advanced host‐profiling technologies with shotgun metagenomics, and untargeted metabolomics will allow us to link specific microbiota‐produced metabolites to phenotypes of individual immune cells to understand precisely why a sub‐optimal immune response to vaccination occur.

Furthermore, given the established safety profiles of many vaccines and microbiome‐targeted interventions, experimental medicine studies in humans offer an exciting prospect. Combining the rigorous design of a RCT, together with advanced multi‐omics technologies may allow for establishing a causal relationship between the microbiome and vaccine immunogenicity in humans while also identifying putative mechanisms. Likewise, we should prioritise a systematic and comprehensive assessment of different microbiome‐targeted interventions to determine the optimal approach to enhance vaccine immunogenicity. Although there have been a number of evaluations of probiotics, these studies have to date focused on traditionally‐used probiotics such as *Lactobacillus* and *Bifidobacterium* as opposed to ‘next‐generation probiotics’ such as *Faecalibacterium prausnitzii* that have arisen from more advanced culturing, sequencing, and genome editing technologies and are postulated to better colonise the human gastrointestinal tract and have more potent effects on the immune system [162, 163]. Finally, another key knowledge gap to be investigated is the role of the non‐bacterial microbiome (archaea, viruses, microbial eukaryotes) in regulating vaccine immunogenicity. With few exceptions [48], this is an area that is unexplored. Given the well‐established role that microbial eukaryotes can play in immune regulation [164, 165] it would seem plausible that they also may impact vaccine immunogenicity. Similarly, the gut virome would seem an obvious source of potential cross‐reactive antigens for viral pathogens given its density (~ 10^9^ virus‐like particles per gram of faeces) and reports of convergent evolution among viral capsids [166].

In conclusion, the COVID‐19 pandemic has spurred a growing appreciation for the complexity of vaccine immune responses. To prepare for the next pandemic, and better combat endemic diseases, it is imperative we understand what drives inter‐individual variability in vaccine immune responses and that we develop strategies to overcome this variability to promote optimal protection.

## Conflict of interest

DJL and FJR have received funding from GSK related to this area of research.

## Author contributions

All authors contributed to the writing, editing, discussion and review of the manuscript.
